# The matrix-dependent 3D spheroid model of the migration of non-small cell lung cancer: a step towards a rapid automated screening

**DOI:** 10.3389/fmolb.2021.610407

**Published:** 2021-03-25

**Authors:** Evgenya Y. Shabalina, Ekaterina Yu Skorova, D. A. Chudakova, V. B. Anikin, I. V. Reshetov, O. A. Mynbaev, E. V. Petersen

**Affiliations:** ^1^ Moscow Institute of Physics and Technology, Institutskiy Pereulok, Dolgoprudny, Russia; ^2^ School of Biological Sciences, University of Auckland, Auckland, New Zealand; ^3^ Brunel University London, Uxbridge, United Kingdom; ^4^ First Moscow State Medical University, Moscow, Russia

**Keywords:** 3D cell culture, multicellular tumor spheroids, non-small cell lung cancer, metastasis, microscopy

## Abstract

*In vitro* 3D cell culture systems utilizing multicellular tumor spheroids (MCTS) are widely used in translational oncology, including for studying cell migration and in personalized therapy. However, early stages of cellular migration from MCTS and cross-talk between spheroids are overlooked, which was addressed in the current study. Here, we investigated cell migration from MCTS derived from human non-small cell lung cancer (NSCLC) cell line A549 cultured on different substrates, collagen gel or plastic, at different time points. We found that migration starts at 4–16 h time points after the seeding and its speed is substrate-dependent. We also demonstrated that co-culture of two NSCLC-derived MCTS on collagen gel, but not on plastic, facilitates cell migration compared with single MTCS. This finding should be considered when designing MCTS-based functional assays for personalized therapeutic approach and drug screenings. Overall, our work characterizes the *in vitro* 3D cell culture model resembling NSCLC cell migration from the clusters of CTCs into surgical wound, and describes microscopy-based tools and approaches for image data analysis with a potential for further automation. These tools and approaches also might be used to predict patterns of CTCs migration based on *ex vivo* analysis of patient biopsy in a 3D culture system.

## Introduction

Lung cancer (LC) is the most common type of cancer, and non-small lung cancer (NSCLC) is the most common type of LC, accounting for approximately 85% of LC cases (Aisner and Marshall, 2012; Rivera et al., 2013). Surgical resection of the tumor is the therapy of choice for patients with early stages of LC (Lang-Lazdunski, 2013; Uramoto and Tanaka, 2014). However, the secondary metastatic tumor can arise as a result of the surgery and surgical stress (Tohme et al., 2017), and dissemination of the cancer cells after tumor resection can result in metastasis and formation of the secondary metastatic tumor (Grotzer et al., 2016). The metastatic cascade is a multistep process, starting from the malignant transformation and growth of the primary tumor, local invasion of the surrounding tissues by the cells, dissemination of the circulating tumor cells (CTCs) into the circulatory system, and finally invasion and colonization of the secondary tissue by the disseminated cells originating from the primary tumor (Eslami et al., 2020; Lozar et al., 2019). CTCs can travel in a bloodstream as a single cell or a cluster of the cells (also known as “circulating tumor microemboli,” CTM), and the latter appear to have higher metastatic potential (Micalizzi et al., 2017) supposedly due to the changes in DNA methylation promoting stemness (Gkountela et al., 2019). Epithelial-to-mesenchymal transition (EMT) resulting in loss of epithelial markers and gain of migratory mesenchymal-like phenotype and enhanced invasiveness is one of the key contributors to the metastatic cascade (Ribatti et al., 2020; Samatov et al., 2013). However, while some cells within the cluster of CTCs undergo EMT (Genna et al., 2020), it is still not a critical and ultimately necessary event for CTCs engraftment and target tissue invasion, and there are non-EMT-mediated mechanisms of invasion described (Chen et al., 2017). In case of post-surgical secondary tissue invasion by the CTCs, they do not necessarily express EMT markers and do not need to acquire mesenchymal phenotype to detach from the primary tumor. It seems reasonable to suggest that risk of the post-operational secondary metastatic tumor, at least partially, is associated with the increased numbers of the CTCs clusters generated by the surgical resection of the tumor tissue, which has been reported for several types of cancer including LC (Duan et al., 2019; Chudasama et al., 2017; Matsutani et al., 2017), although other mechanisms, such as perioperative activation of the dormant metastatic cell clusters in the host microenvironment, and others also play a role. In either scenario, migration of the cancer cells is a prerequisite of metastasis and is orchestrated by the TME (Gay and Malanchi, 2017). Notably, in NSCLC there was no association between CTCs and long-term survival of the patients observed (Barr et al., 2020). While mechanisms of “classical” metastasis cascade are well-studied (Dianat-Moghadam et al., 2020), there is a gap of knowledge of the mechanisms of post-surgical invasion of the secondary tissue by the CTCs, as current knowledge about CTCs biology is very limited (Mathias et al., 2020). The mechanisms of the post-surgical CTCs wound implantation and local migration into the secondary tissue (often not involving entry into the bloodstream) potentially leading to the metastasis within the surgical scars are undoubtedly different compared to “classical” metastasis, because of the alteration in the composition and structure of the surgically damaged target tissue (for example, disrupted basal membrane), the influence of the post-surgical inflammatory milieu, as well as specific phenotypic characteristics of the post-surgical CTCs clusters (for example, difference in the level of expression of EMT markers by CTCs, stromal cell composition within the CTCs, and others). As damaged tissue might be more permissive for CTCs invasion, preventing early postoperative spread of the CTCs is of particular importance (Alieva et al., 2018), especially during the therapeutic window between the surgery and subsequent chemo- or radiotherapy. This strongly advocates for perioperative therapeutic options targeting CTCs (Mitchell et al., 2014) and tumor cell migration, thus *in vitro* cell models mimicking migration of the cancer cells from the CTCs clusters into ECM would be a valuable tool in translational oncology and clinic, allowing for personalized therapeutic approach and drug screening.

Hitherto, many cell-based models simulating attachment, migration and tissue-invasion of the cancer cells exist (Simeone et al., 2019; Rodrigues et al., 2020; Ko et al., 2021). The most physiologically and clinically relevant models are 3D cell culture models resembling architecture of the cluster of CTCs and incorporating some elements of the tumor micro-environment (Kunjithapatham et al., 2014; Caleb and Yong, 2020). For example, several systems for CTCs culture, including 3D monoculture of CTCs on a gel, have been established for patient-derived LC cells (Zhang et al., 2014).

Multicellular Tumor Spheroids (MCTS), resembling clusters of cancer cells, is one of the most commonly used 3D cell culture models in cancer cell migration research, valuable tool in personalized medicine when based on patient-derived cells (Fong et al., 2017), and a system instrumental in anti-metastatic drug screening (Edmondson et al., 2014; Vinci et al., 2013), although it should be noted that cancer cell migration is “a requisite, but does not necessarily predict metastasis” (Popper, 2020). There are several important points which need to be addressed while modeling cell migration from CTCs clusters. The process of tumor cell migration is profoundly influenced by the tissue microenvironment (TME), including extracellular matrix (ECM), stromal cells, and other components of TME (Winkler et al., 2020), thus *in vitro* systems which recapitulate tumor cell migration and tissue invasion should incorporate ECM and assess its role in cellular behavior. Moreover, most of the conventional *in vitro* models of cancer cell migration are monolayer 2D cultures which are less physiologically relevant than 3D cultures (Jensen and Teng, 2020). Many of *in vitro* 3D culture models (including those based on of non-small cell lung cancer cell line which was used in our study) assess migration of the cells only at the later time points, 24–48 h after cell seeding, while initial stages of cell outgrowth and migration are overlooked (Miyazaki et al., 2019; Kumari et al., 2020). Finally, an automation of the analysis of the cellular behavior in such model systems would bring several advantages in the clinic and in translational research, decreasing cost and time of the test.

To address all of this, we applied microscopy-based analytical tools which have the potential to be automated to characterize models of early stages of cell migration from CTCs clusters utilizing non small cell lung cancer.

## Materials and Methods

### Cell Culture and Spheroid Formation

Human NSCLC cell line A549 was cultured in DMEM/F12 medium containing 1% Penicillin Streptomycin (Thermo Fisher Scientific), 1% Insulin-Transferrin (Thermo Fisher Scientific), and supplemented with 10% Fetal Serum Albumin. Cells were cultured at 37°C in a humidified atmosphere containing 5% CO_2_. MCTS were generated as described previously (Ilhan-Ayisigi et al., 2020), with slight modifications. The MicroTissues® 3D Petri Dish® micro-mold spheroids, size L, 5 × 7 array (Sigma-Aldrich) was used to generate MCTS. Each MCTS consisted of approximately 200 cells; the radius of the MCTS was 135 ± 10 μm. For MCTSs cultivation on ECM-mimicking substrate neutralized collagen I gel with final concentration 10 μm/ml was used with the final thickness of prepared gel 0.94, 1.6, and 3.2 mm using a slightly modified protocol from Kuczek et al. (Kuczek et al., 2019).

### Resazurin Metabolic Activity Assay

The metabolic activity of the cells was measured using resazurin-based assay, following standard protocol as described previously (Ivanov et al., 2014). The excitation/emission of the samples was measured at 550/585 nm, correspondingly, using a Plate Reader CLARIOstar.

### Microscopy and Image Analysis

Multiple images of the cells were acquired using Zeiss inverted microscope (Axiovert 200M). Images were analyzed in ImageJ image processing and analysis program (https://imagej.nih.gov/ij/). Average radius of migration and contact area of migrating cells were calculated as follows. The counting was carried out from the center of the spheroid. For each spheroid, five measurements of the radius of cell migration from the spheroid body were performed and then averaged. The area of the spheroid was determined manually as the visible border of the outer cells of the migrating front. For each time point at least three spheroids were analyzed. The average speed of cell expansion/migration rate was calculated as angular coefficient of linear approximation of the curve representing dependence of the radius on time. The linear approximation coefficients were determined by the least squares method with the determination coefficient *R*^2^.

For optical density analysis of the image eight radii were drawn from the center of each spheroid, next the average intensity of the gray color was calculated over a segment equal to half the width of the corresponding zone S0, S1, S2 and half the width of the S2 zone in the background, thus data from 33 points were obtained for each of eight spheroids for every time point. Average values were calculated for each zone with error bars representing standard deviation values (SD). By average deviation we mean the average linear deviation, which is the average of absolute values of data point deviations from the average. The following time points were selected for analysis: 2, 4, 6, 8, 12, 16, 17, 24, and 36 or 48 h from the moment the spheroids landed on the matrix.

Cell viability was assessed by staining with 2′,7′- dichlorodihydrofluorescein diacetate (H2DCFDA, Fd) (Invitrogen) using the vendor recommended protocol.

### xCELLigence Real-Time Cell Analysis

MCTS were settled in pre-warmed E-plates and incubated in xCELLigence RTCA system (Agilent) at 37°C in a humidified atmosphere in presence of 5% CO_2_ for 20 h. Quantified changes in the size of the spheroid and the area of the migrating outgrowth cells over the time course were analyzed with an integrated software package (error bars represent SD), at least two runs with no less than six repeats were performed for each experiment.

### Statistical Analysis

Statistical analysis was performed in Microsoft Office Excel software using built-in functions (Student’s t-test, linear approximation). Data are presented as mean ± Standard Deviation (SD). Comparison between MCTSs cultured under different conditions was performed using Student’s t-test with GraphPad Prism 8 (United States). *p*-values lower than 0.05 were considered significant.

## Results and Discussion

The NSCLC adenocarcinomas are very prone to metastasis, including post-surgical metastasis induced and facilitated by the tumor resection and dissemination of the CTCs clusters. In our study we used NSCLC adenocarcinoma cell line A549 to establish and characterize 3D multicellular spheroid model of cell migration from the CTCs cluster (engraftment of CTCs and migration of the cells from CTCs into the new niche). Previous studies utilizing 2D culture of this cell line and focusing on metastasis-like migration have demonstrated that it can be pharmacologically inhibited (Kang et al., 2020; Liu et al., 2013). However, it remains unclear whether these data will be reproduced in the 3D culture systems, which are more physiologically and clinically relevant. Therefore, 3D culture models of NSCLC metastasis-like migration, as well as tools and approaches to analyze them are needed, which was addressed in the current study.

**Cell migration from the MCTS is an early event, and the patterns of cell migration from MCTS on plastic and on collagen gel are different.**

**FIGURE 1:**
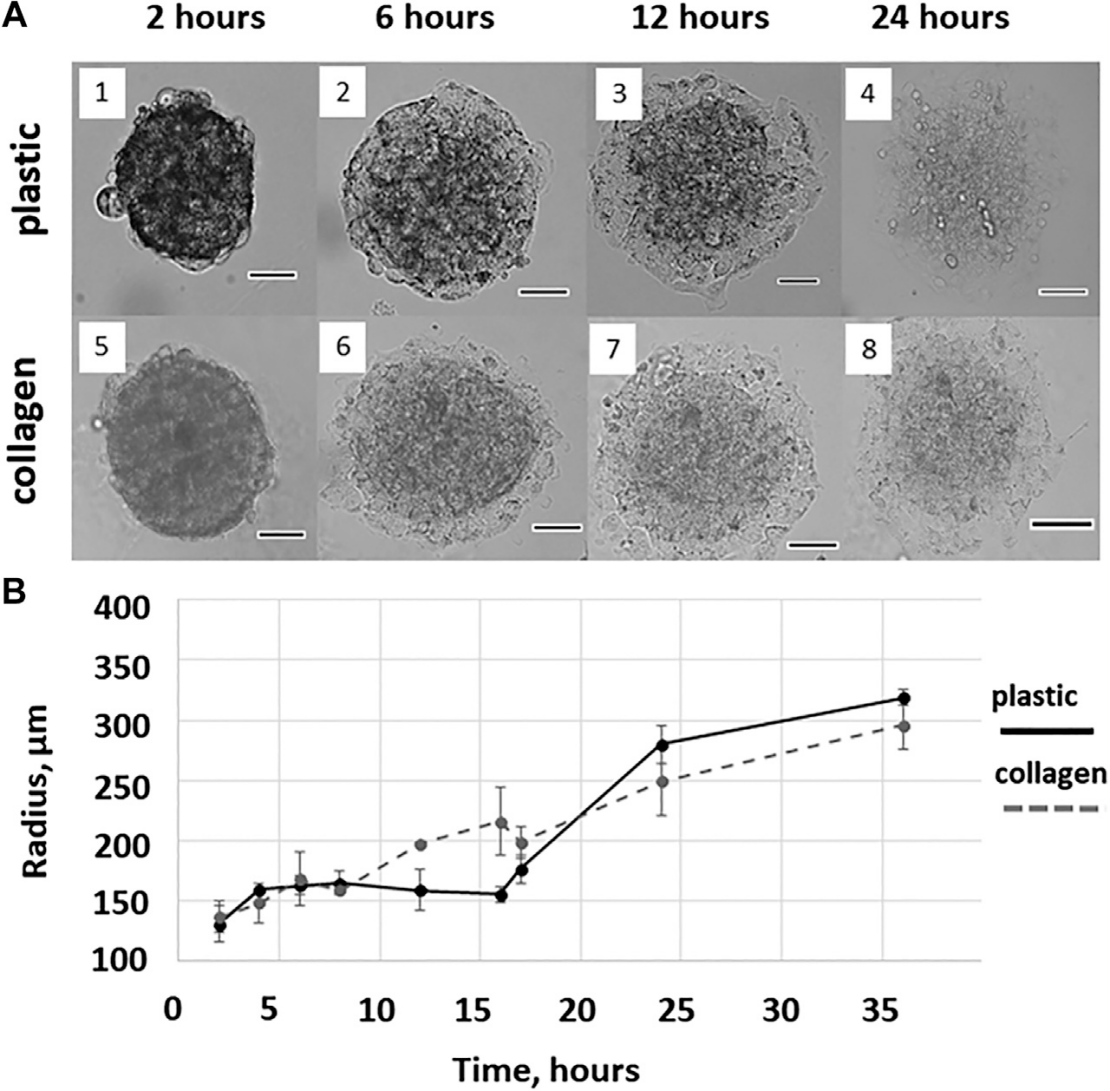
Cell migration from the MCTS on plastic and collagen gel. **(A)** Representative images of MCTS cultured on plastic or collagen for 2, 6, 12, and 24 h (1,2,3,4 and 5,6,7,8, respectively). Scale is 100 μM **(B)** The mean radius covered by the cells migrating from single MCTS on plastic or collagen gel. The curves represent combined data from two independent experiments and three to nine technical replicates per time point. The data are presented as the mean ± SD.

One of the possible models of metastasis is a transformation of MTCS into the cellular monolayer (Kunjithapatham et al., 2014), or migration of the cells from the MTCS. It is a well-known fact that the ECM affects cancer cells migration and tissue invasion, thus in our model we assessed the difference in cell migration from MCTS cultured on different substrates. Here, we characterized several stages of the cell migration from the MCTS on plastic or collagen substrate, starting from 2 h time point (Figure 1; Supplementary Figure S1). The microscopy and imaging analysis demonstrated that migration of the cells from MCTS starts as early as 6 h (Figure 1A2,6). This suggests that therapy targeting post-operative metastasis should be applied as soon as possible after the tissue resection. Also, in personalized medicine this time frame should be used while assessing in *ex vivo* models the response of the patient’s tumor cells to the anti-metastatic chemotherapy and their patterns of migration.

We also found a difference between the mean radius covered by the cells migrating from the MCTS on plastic and collagen gel, which was observed as early as at a time point of 12 h and started disappearing after 16 h (Figure 1B).

This allows us to speculate that after the first 16 h of culturing the difference between the seeding matrices (collagen gel or plastic) is eliminated because cells seeded on plastic produce and secrete enough of ECM molecules. This may also explain the fact that during the first hours of culturing cells from plastic-seeded MCTS migrate much slower than those from collagen-seeded MCTS, since they have yet to secrete ECM molecules facilitating migration. Since collagen gel is biologically active ECM, cells from collagen-seeded MCTS do not have to secret growth factors and ECM components to migrate, therefore there is no initial period of slow cell expansion in case of collagen-seeded MCTS.

We also analyzed the process of cell migration from the MCTS using the xCELLigence real-time cell analysis (RTCA) method, determining the area of the cellular contact with the substrate and change of this area over the time.

**FIGURE 2:**
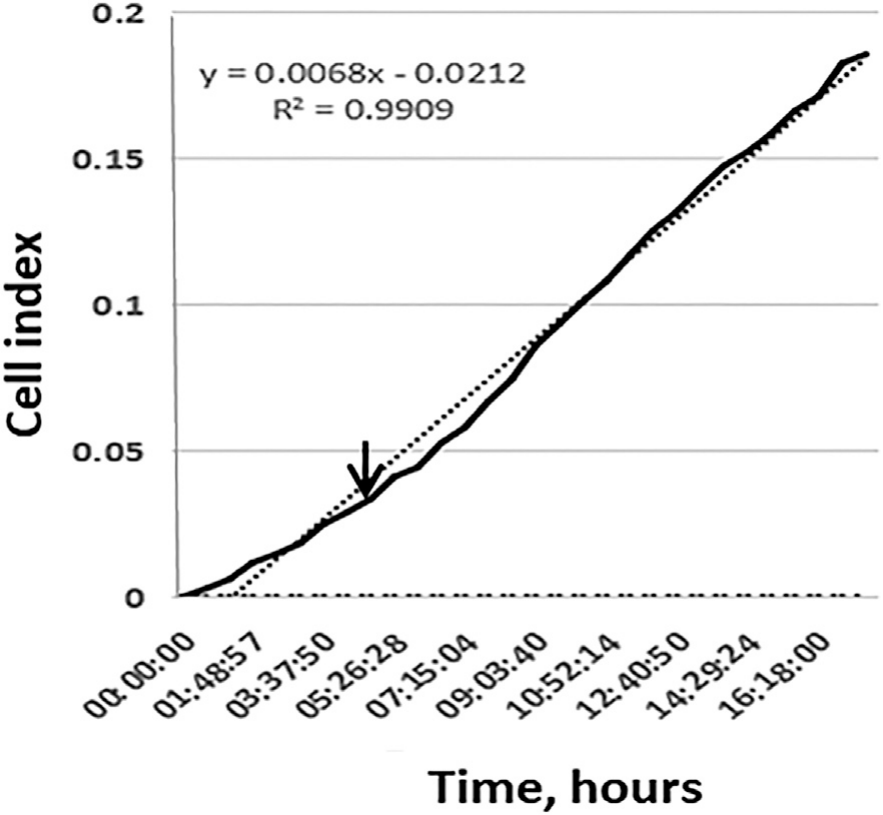
The active migration of cells from MCTS seeded on plastic starts at time points 4–16 h. Representative image of the kinetic data for cell migration from single MCTS seeded on plastic generated by xCELLigence RTCA system. Dotted line represents a linear trend of the curve, dashed line corresponds to the background signal from the empty well. Expansion speed increases and overtakes the trend line at time point 3–4 h (indicated by arrow).

The RTCA method takes into account the uneven migration of cells from the body of the MCTS in different directions and allows a more accurate assessment of the migration process. In Figure 2.

**FIGURE 3:**
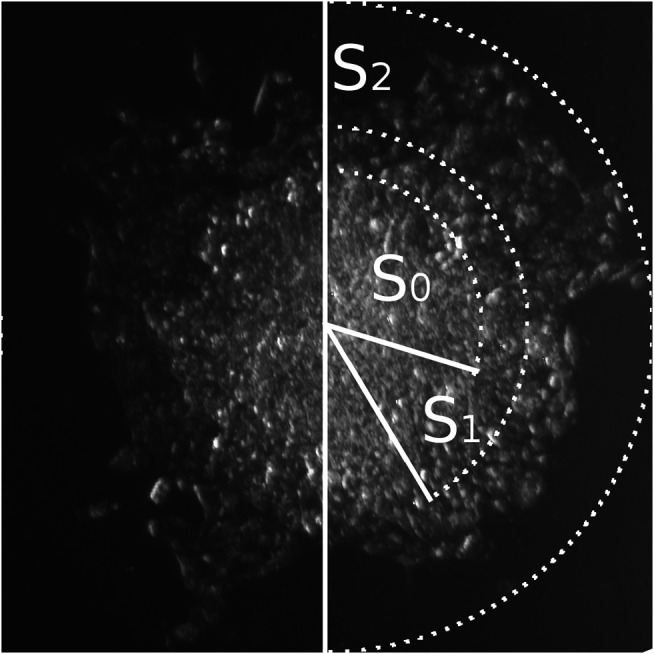
Three zones of cellular migration from MCTS. Representative image of the MCTS on plastic at the time point 24 h with zonal segmentation to S0, S1, and S2 areas characterized by different optical density of cell layers.

We demonstrate that during the first 10 h expansion speed of single MCTS seeded on plastic (shown in black) is lower than mean expansion speed during 20 h of experiment (dotted trend line). Later the expansion speed increases and overtakes the trend line that in turn leads to an s-shaped curve formation. These results are in line with the data shown in (Figure 3C). Change over the time in the area which is occupied by cells demonstrates that during the first 4 h after the seeding of MCTS the rate of area growth was relatively lower compared to those of later time points (4–16 h), which means that active migration of cells from MCTS starts at time point 4–16 h (Figures 3C,D). At the time interval of 16–24 h the expansion speed reaches its maximum and exceeds the calculated mean values both in RTCA assay and when measured by radius or area. The association between the patterns of migration and metabolic activity of the cells was also observed (Supplementary Figure S1A).

**There are three distinct zones of cellular migration from MCTS.**

We further focused on using the mean area parameter, since it can be rather easily automatically generated from the images through the binarization algorithms and therefore is more suitable for the analysis. Such analysis applied to the microphotography data can be automated, as discussed below, allowing for rapid high-throughput analysis of the big scale experiments. Using this approach, we have identified three distinct zones of cellular migration from MCTS (Figure 3), most clearly distinguishable at late time points (12 h and later), and utilized microscopy analysis to measure the optical density of the cells in those zones and characterize the patterns of their movement.

**FIGURE 4:**
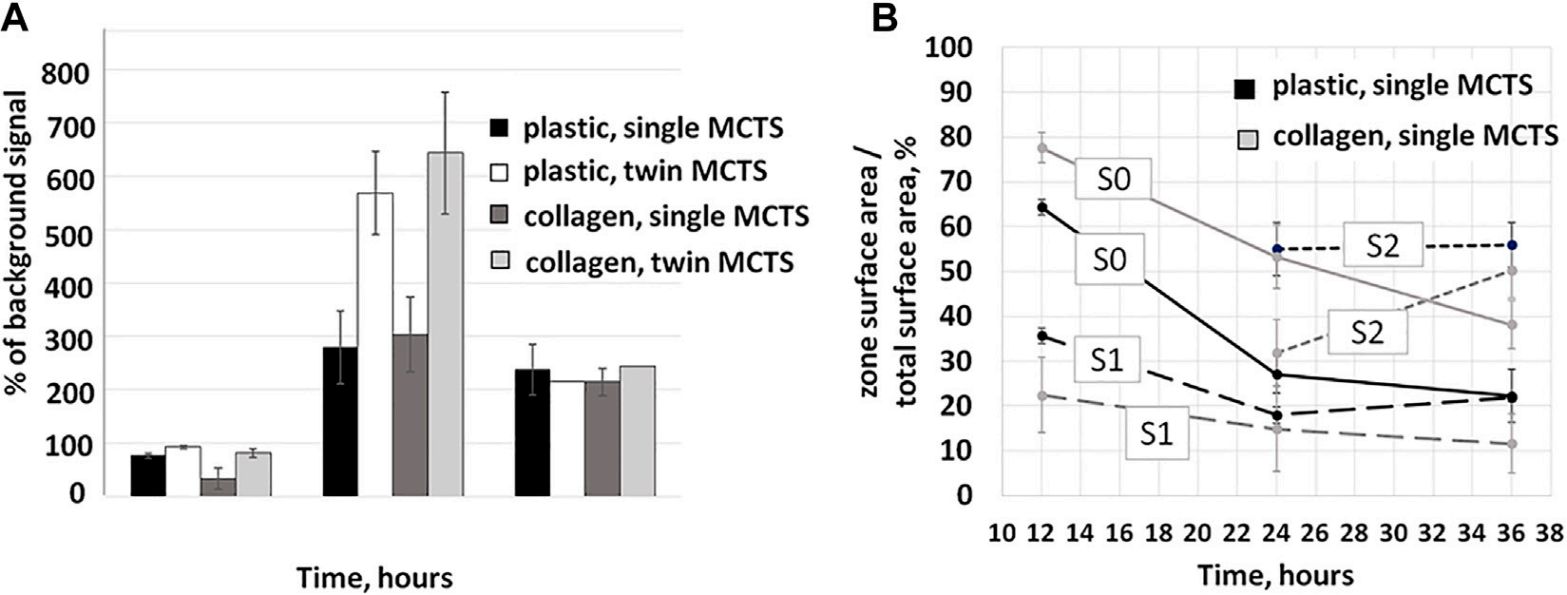
Changes in metabolic activity and area share of single and twin MCTS in the first 36 h of culturing. **(A)** Resazurin assay was used to assess metabolic activity of the cells. Increase of the fluorescence ratio relative to background values demonstrates an increase in metabolic activity. Data obtained from two experiments with three to nine technical repeats **(B)** Time-dependent changes in migration zone area shares of the total area of the MCTS. Data obtained from three repeats and presented as the mean ± SD. dynamically change over the time course.

The first zone of cellular migration from MCTS, S0, corresponds to the “body” of the MCTS. S1 is the “inner ring” of the cells migrating from the MCTS; this zone is characterized by a high cellular density. “Outer ring” S2 is a zone with normal cellular density formed by the cells which are completely spread over the substrate and seem to actively migrate and divide (Figure 3; Figure 4B). Overall, we have identified three distinct zones of the cell migration from MCTS which the surface area-based analysis shows that the S0 plot decreases over the time. This is due to the fact that after 12 h the cells begin to migrate out of the MCTS body, thus while the total area covered by the cells increases over the time, the share of S0 in the total area decreases. The area of S1 did not change significantly over the time course. This suggests that cells from this zone do not move much or spread over the substrates, regardless of whether it is collagen or plastic substrate. The share of this zone also decreases over time. The highest rate of migration and increase in the zonal area is characteristic of the cells of the outer ring, S2. The starting point of the graph corresponding to the S2 zone (Figure 4B) is 24 h, because only starting from this time point the S2 becomes distinguishable from the S1 zone. The changes in the size of the zone surface area over the time for collagen-seeded MCTS and for plastic-seeded ones have a common pattern.

The body of literature demonstrates that cancer cell metabolism affects migration and invasion of the tumor (comprehensively reviewed in (Han et al., 2013)). Thus, we expect to observe such a relationship between the metabolism and migration in a physiologically relevant model of cancer cell migration. In our study, the association between the patterns of migration and metabolic activity of the cells was also observed (Figure 4A).

**FIGURE 5:**
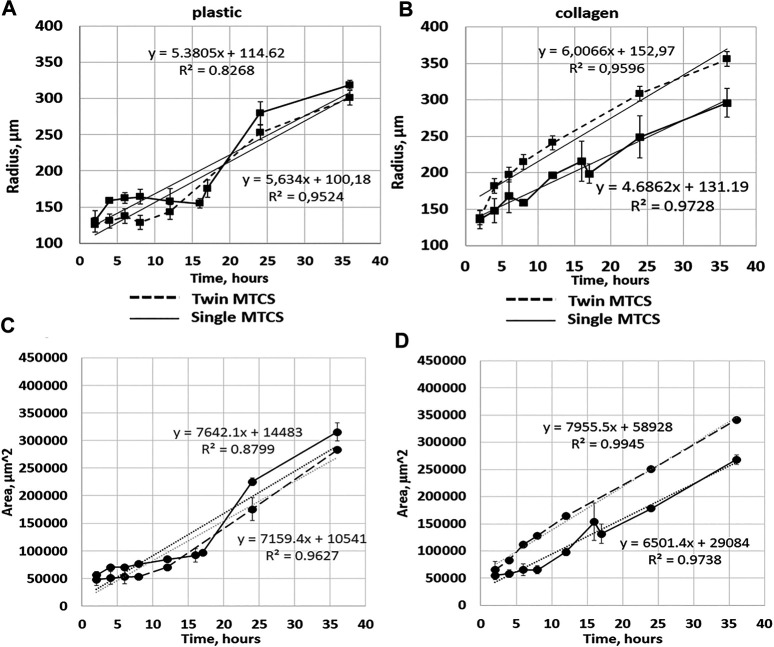
Cell migration from single MCTS and twin MCTS on plastic and collagen gel. **(A)** Average radius of cell migration from single MCTS and twin MCTS planted on plastic substrate **(B)** Average radius of cell migration from single MCTS and twin MCTS planted on collagen gel. **(C)** Average area of cell migration from single MCTS and twin MCTS planted on plastic substrate **(D)** Average area of cell migration from single MCTS and twin MCTS planted on collagen gel. The data are presented as the mean ± SD. Linear trend (dotted line) represent mean expansion speed over 24 h with trend parameters shown in the graph. Corresponding data values are shown in Supplementary Tables S1, 2, 4. expansion speed so drastically (5,385 c. u. vs 5,634 c. u. for single and twin MCTS, respectively (Figure 3, Supplementary Table S4)), especially in the early stages.

The maximum metabolic activity of the cells was detected at the time point of 24 h for both single and twin MCTS on any substrate (Figure 4A). At the time point of 36 h after the seeding, the metabolic activity of the cells, as measured by the intensity of the fluorescence in the culture media, dropped. This coincides with the decrease in the rate of the cellular migration, based on an estimate of the covered surface area at the corresponding time points (Figure 5). Our data demonstrates that in terms of metabolic activity twin MCTS behave differently compared to single ones (Figure 4A). At the time point of 12–24 h metabolic activity in twin MCTS is two times higher compared to the single ones, on both substrates. These doubled rates of metabolic activity in twin MCTS could be explained simply by the double number of cells in twin MCTS compared to single ones. Moreover, there was no change in behavior of the cells as estimated by the measurement of the mean radius and mean area values of the MTCS at the same time points (Figures 5A–D). Yet, at the time point of 24–36 h we could not detect any significant difference in metabolic activity between single and twin MTCS, as measured by the resazurin assay, although there still was about twofold difference in total cell number between single and twin MTCS. We suggest that the possibility of fast metabolic shifts during first hours of migration and their connection to the increase in expansion area should be further examined by methods that are more sensitive then resazurin assay, which was beyond the scope of the current study. Overall, we have identified three distinct zones of the cell migration from MCTS which dynamically change over the time course.

**There is a possibility for the automation of the data analysis in this model.**

The automatic image quantification and analysis in 3D cell culture models of cancer cell migration would be of obvious advantage in translational oncology, therefore several approaches allowing for it are currently being developed (comprehensively reviewed in (Driscoll and Danuser, 2015)). Works focusing on the automated models of cell migration segmentation also exist. For example, a semi-automated model of segmentation of neuroblast migration was presented recently (Ducker et al., 2020). This model, while being a powerful tool, has some limitations, for example in order to estimate the area of migration, the criteria was used when ROI “intersected at least six cells deemed to have migrated from the spheroid,” which may actually refer to the maximum radius of the migration area rather than the area populated by the cells. Thus, the question of how to reliably perform an automated assessment of the area occupied by the cells migrating from MCTS remains open. In our study, firstly we manually analyzed bright-field images of MCTS in ImageJ. Since there was a convincing similarity of the results obtained by calculating expansion rates through mean radius or mean area (Figure 5), we chose to use mean area parameter as a characteristic for the rate of the cellular migration from the MCTS body. As discussed earlier, we find the mean area parameter to be more suitable for the analysis, since it can be automatically generated from the images through the binarization algorithms, which are commonly used for image analysis (Bernsen, 1986). This in turn can be used for further automation of the data analysis in the high-throughput screening systems based on the MCTS models.

**FIGURE 6:**
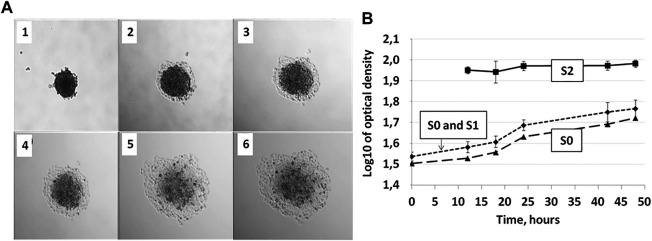
Zones S0, S1, and S2 during 36 h of incubation of the MCTS on plastic are distinguishable by optical density allowing for automated image analysis. **(A)** MCTS on plastic immediately after planting (1), after 12 h (2), 18 h (3), 24 h (4), 28 h (5), and 48 h (6) of culturing **(B)** Changes in the relative values of gray color in S0, S0, and S1 and S2 zones of the cell inhabited area. Data obtained from eight technical repeats and presented as the mean ± SD. For corresponding values see Supplementary Tables S1, 2, 4.

As we discussed previously, the patterns of the S0-S2 segmentation vary depending on the metabolic activity of the cells and can be a biomarker of such activity. While analyzing microphotographs by the level of optical density we found that it is possible to distinguish the zones S0, S1 and S2 based on the intensity of the gray color (optical density) in the corresponding area—the optical density of the cells in zone S2 is significantly different from the areas S0 and S1 (Figure 6). The optical densities of the cells in S0 and S1 are statistically indistinguishable, which can be explained by the high density of cells in the S1 zone. Such image analysis can be automated.

**The patterns of cell migration from the single MCTS and from the two MCTS cultured in close proximity to each other are different on collagen gel, but not on plastic.**

It has been previously shown that the number of the CTCs clusters circulating in the bloodstream significantly rises following surgical resection of LC tumor (Duan et al., 2019), increasing the chance of the two CTCs ending up attached to the ECM in close proximity to each other. It is also known that cancer cells can change ECM creating metastasis-permissive niche (Zhuyan et al., 2020), and therefore its plausible to suggest that cooperative modification of the ECM by several clusters of the cancer cells located close to each other can facilitate their migration. The existence of cancer-promoting cross-talk between cancer cells, stromal cells and tumor microenvironment is well known (Liu and Cao, 2016) and undoubtedly accepted, hence it would not be surprising if communication between CTCs within the target tissue also existed and resulted in enhanced tumorigenesis and metastasis. Thus, we investigated whether co-culture of MTCS in close proximity to each other can affect migration of the cells from MCTS and, hence, metastasis.

We found that cell expansion speed (angular coefficient of linear approximation of the curve) was higher on collagen gel in case of “twin MCTS” (two MCTS cultured in close proximity to each other) compared to single MCTS (4.686.2 c.u. for single vs 6,006.6 c.u. for twin MCTS (Figure 5; Supplementary Table S4)). In case of MCTS seeded on plastic, twin cultivation did not affect cell.

**FIGURE 7:**
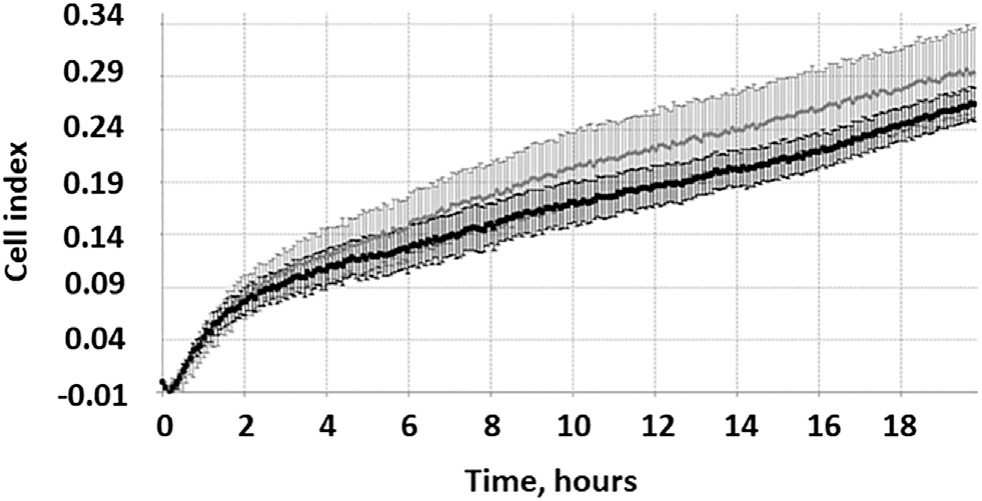
RTCA measurements of growth kinetics of the area inhabited by the cells migrating from single and multiple MCTS on plastic. Representative image of growth kinetics for single MCTS (gray curve) and multiple MCTS (black curve) normalized by control (empty well). The data are presented as the mean ± SD. Representative data from two of 12 repeats in two runs.

The average rate of change in the mean contact area of spheroid cells seeded on plastic was 7,641 c.u. for single MCTS and 7,149 c.u. for twins. For MCTS seeded on the collagen gel, these values were 6,502.2 c.u. for single and 7,931.4 c.u. for twins, respectively, which is a noticeable difference (Figure 5). The change of the radius of the area covered by the cells over the time was somewhat similar in case of single and twin MCTS cultured on plastic, but different in case of MCTS cultured on collagen gel (Figures 5C,D). Thus, we found that the migration patterns of the outgrowth cells from the single MCTS and twin MCTS on the collagen gel were different, suggesting a cross-talk between the MCTS either via the secretion of regulatory molecules or via the modification of the collagen matrix. At the same time, there was no significant difference between the behavior of the cells migrating from the single or twin MCTS on plastic. Therefore, the differences in migration patterns are most likely linked to the interaction of MCTS with the matrix and the characteristics of the matrix. This was confirmed by results of RTCA analysis demonstrating that on plastic the area occupied by cells does not depend on the number of MCTS per well and always grows according to the similar trend, even if the number of MCTS was increased to 15 per well (Figure 7).

It should be noted that RTCA does not allow us to compare absolute area values therefore we cannot speculate upon significant differences in colonized area between single and multiple plastic-seeded MCTS. Yet we can suggest that there is no qualitative difference between the trends of migration from the single or multiple MCTS on plastic.

To sum up, co-culture of twin MCTS compared with culture of single MCTS facilitates cell migration on collagen gel, but not on plastic. We hypothesize that this phenomena is caused by the cross-talk between MCTS, and interaction of MCTS with the ECM resulting in its subsequent modification creating a migration-permissive microenvironment. At least in part, it might be a mechanism similar to the pre-metastatic niche priming by the primary tumor resulting in ECM alterations (Paolillo and Schinelli, 2019).

**Effect of carboplatin treatment on MCTS in our system can be assessed by RTCA, but not by image analysis.**

**FIGURE 8:**
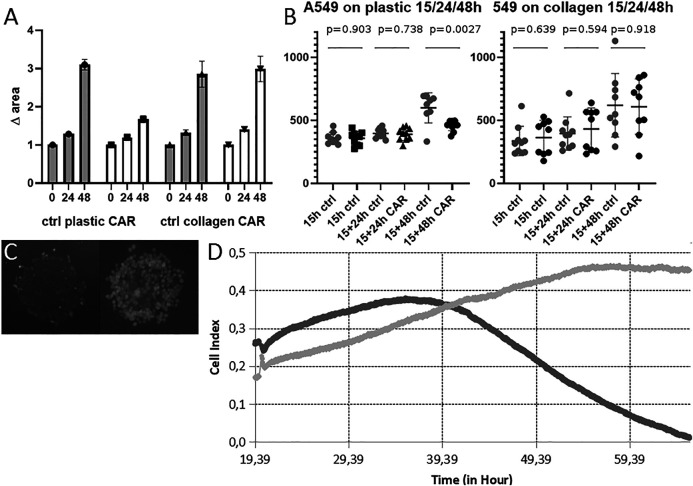
**Cell migration from MCTS on plastic and collagen in presence of carboplatin. (A)** Changes in the area inhabited by cells after incubation with 100 μM carboplatin for 24 and 48 h on plastic and collagen. Carboplatin was added 15 h after MTCS seeding. Data obtained from three technical repeats with 10 wells for each condition and presented as the mean ± SD **(B)** Changes in diameter of MTCS incubated in presence of 100 μM carboplatin for 24 and 48 h on plastic and collagen. Data obtained from three technical repeats and presented as the mean ± SD. Cell viability after incubation with 100uM carboplatin for 24 h followed by 48 h washout on plastic. **(C)** MTCS incubated on plastic in presence of 100 μM carboplatin for 48 h **(left)** and in control **(right)**. Staining with Fluorescein vital dye reveals live cells. **(D)** RTCA measurements of growth kinetics of the area inhabited by the cells migrating from single MCTS incubated with carboplatin on plastic. Representative image of growth kinetics for control MCTS (light gray) and MCTS incubated with 100 μM carboplatin added at 20 h for 48 h (dark gray) normalized by empty well. Representative data from two repeats in six runs.

Next, we assessed whether our system can be used to analyze cell migration in presence of carboplatin—the pharmacological agent commonly used for NSCLC therapy (Green et al., 1992; Tian et al., 2020)—and, thus, potentially in presence of any other pharmacological agent (Figure 8).

As expected, carboplatin inhibited growth of the area inhabited by the cells cultured on plastic (it should be noted that RTCA cannot be used to analyze behavior of the cells on ECM substrates due to the technical limitations of assay) (Figure 8D). We obtained slightly different results from the microscopy data; while in case of MCTS cultured on plastic there was a clear effect of the carboplatin after 48 h, as expected based on previously published data, the cells cultured on collagen were not affected by the presence of collagen in terms of change of diameter of MCTS (Figures 8A,B). It has been previously reported that another platinum-based antitumor compound, cisplatin, extensively binds collagen within the tumor tissue (Chang et al., 2016), which might prevent it from going inside the cell. We suggest that the same might happen in case of carboplatin and find it one of the plausible explanations of the observed effect. It is also consistent with previous works demonstrating that presence of ECM modulates antimigratory and apoptotic effects of some anticancer drugs (doxorubicin) (Georgoulias et al., 2012). Additionally, we measured viability of the cells after incubation with carboplatin, and found that even though most of the cells were dead as a result of the exposure to the carboplatin (Figure 8C left panel), they were still attached to the substrate and therefore often detected by the microscopy and image analysis as indistinguishable from alive cells (we believe that this might be circumvented by additional analysis of optical density (mean gray value) of the image). This leads us to the conclusion that cell death after short-time incubation with compounds might be undetectable by this method on some culturing substrates, and therefore our image analysis approach can be used predominantly for assessment of the biological effect of pharmacological or biological molecules inhibiting, facilitating, or guiding cell migration (thus, resulting in significant increase or decrease of the area colonized by cells, or changes in the pattern/direction of migration). The latter is not only of pure academic interest, but for example might be instrumental in translational oncology for development of so-called “CTCs tumor traps” (systems attracting and trapping CTCs in order to remove them from the circulation) (Najberg et al., 2019). Our system can also be used to search for potential therapeutic targets, as it allows assessing the role of various biomolecules in stimulating cell invasion and migration from the CTCs clusters into the ECM and secondary tissue. As mentioned above, cancer cell migration can be pharmacologically inhibited in 2D culture systems (Kang et al., 2020; Liu et al., 2013), but it’s unclear whether these data will be reproduced in the 3D culture systems. Here, we demonstrate that even incorporating into the cell culture system elements of ECM can dramatically affect cellular response to the treatment. This should be taken into account when designing and interpreting results of drug screening.

Given the heterogeneity of tumor tissue and the patient-specific features of the cell migration, CTCs in different tissue environments or from different patients might vary in their response to the therapy and abilities to engraft and invade a new niche. In personalized medicine the migratory potential of the CTCs from a particular patient can be assessed in *ex vivo* cell culture models, based on our system, allowing predicting migration patterns of the metastasis-initiating cells and their response to the treatment.

Overall, our work demonstrates that the patterns of cell migration from MCTS are substrate-dependent, and such migration is an early event starting as early as 4-6 h after seeding of the MCTS. We describe microscopy-based tools and approaches for analysis of the cell migration from MTCS, able to distinguish several zones of cell migration from the MCTS, which have a potential for further automation for image data analysis using MCTS models. We demonstrate that this system can be used for the characterization of the cellular response to biologically active compounds. For the best of our knowledge, this work is also the first to demonstrate that co-culture of two NSCLC-derived MCTS on collagen gel facilitates cancer cell migration compared to single MCTS.

## Data Availability

The original contributions presented in the study are included in the article/Supplementary Material, further inquiries can be directed to the corresponding author.
