# Supplementary figures and images for: The matrix-dependent 3D spheroid model of the migration of non-small cell lung cancer: a step towards a rapid automated screening
Source: Front Mol Biosci. 2021 Mar 25;8:610407. doi: 10.3389/fmolb.2021.610407 (PMC8378843; doi:10.3389/fmolb.2021.610407)

## Slide 1
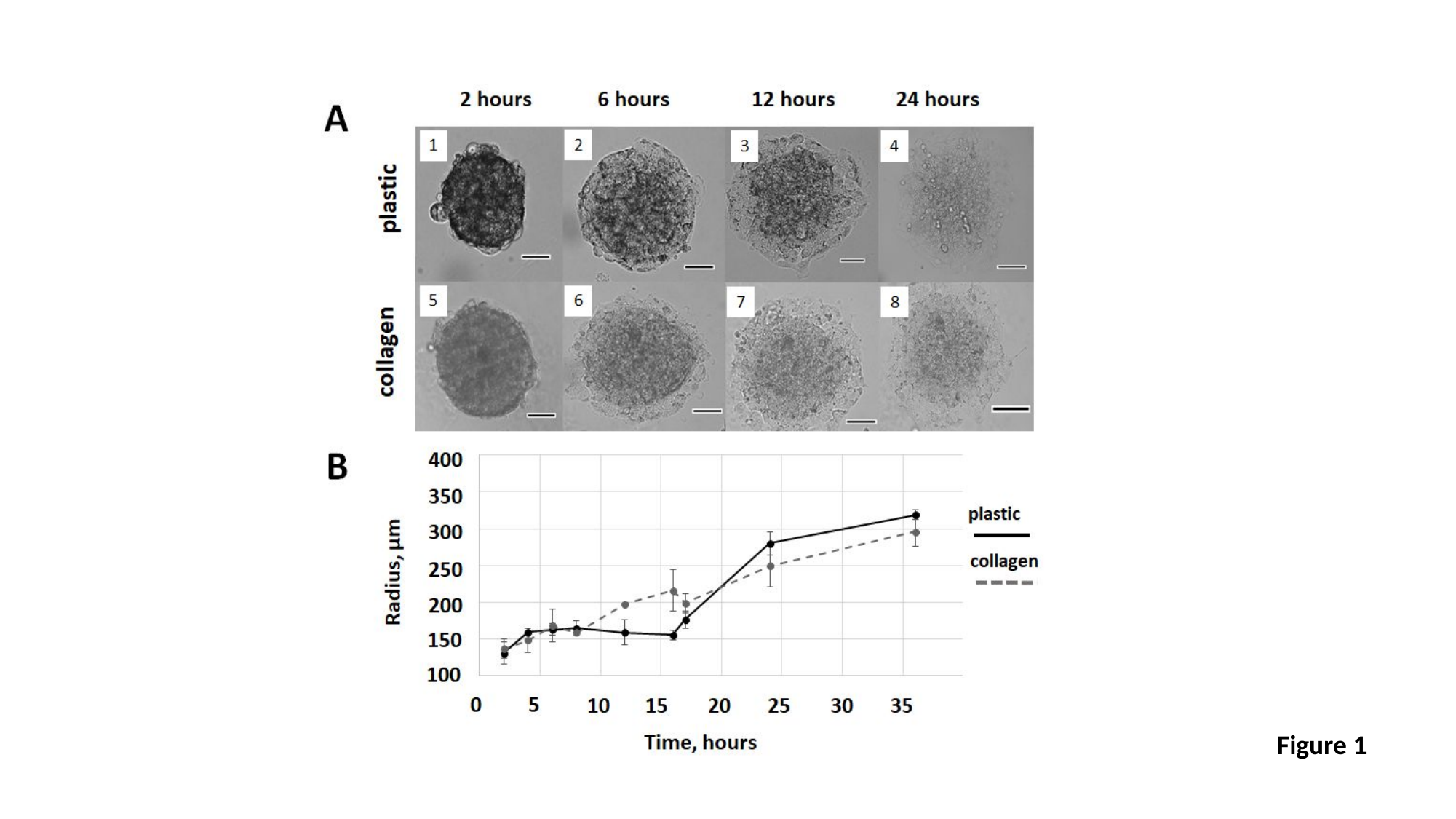

Figure 1

## Slide 2
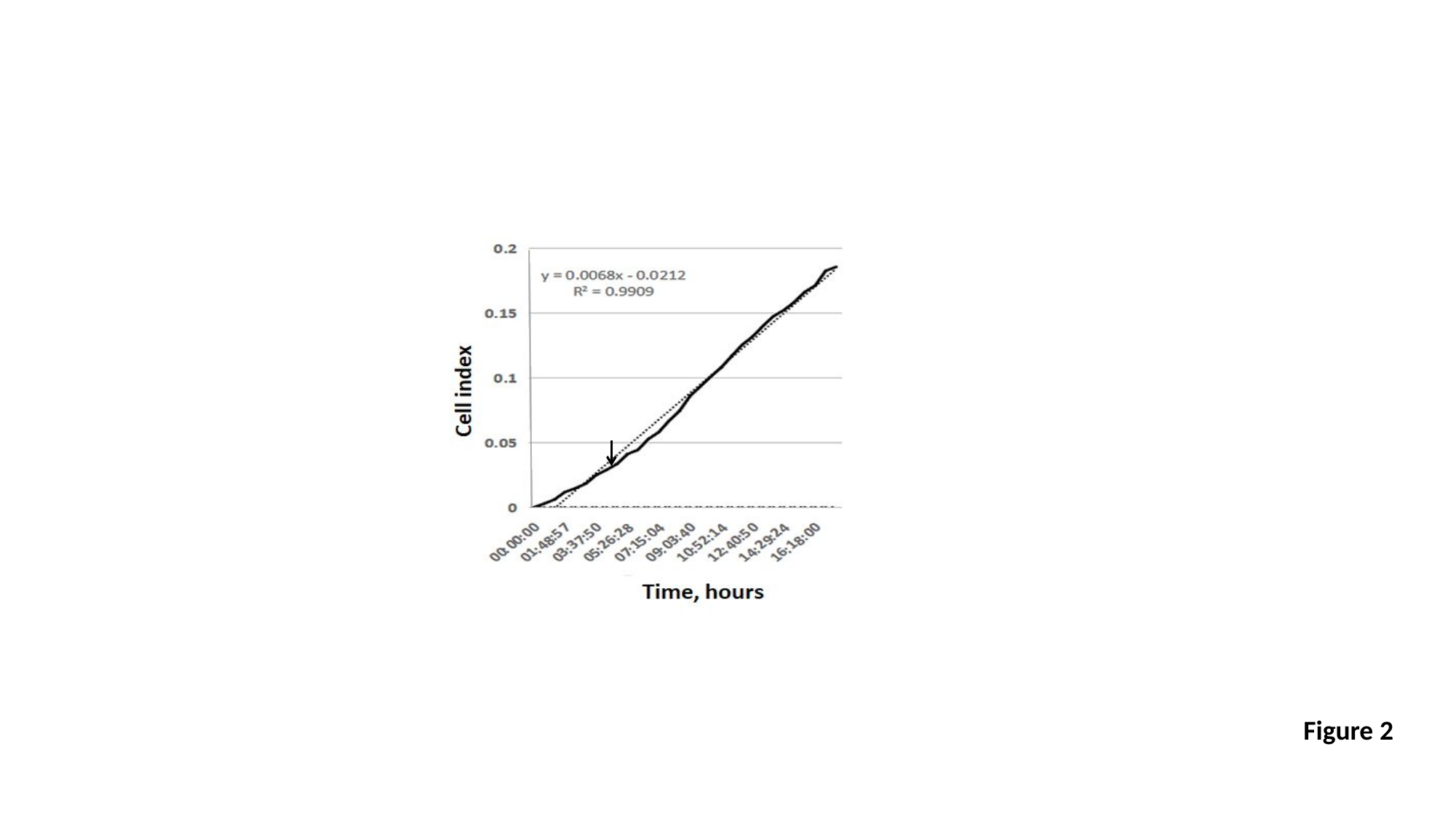

Figure 2

## Slide 3
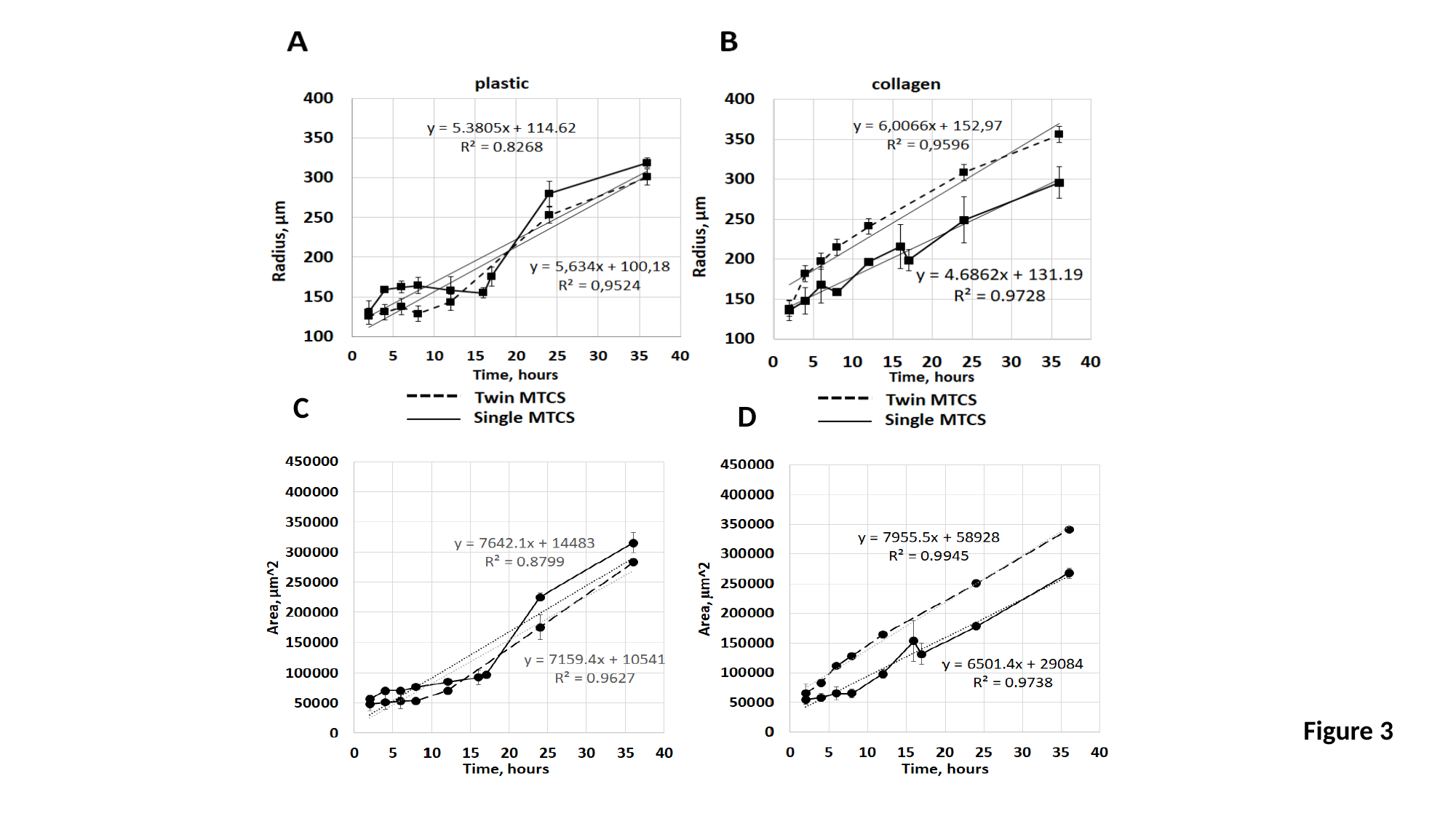

C
D
Figure 3

## Slide 4
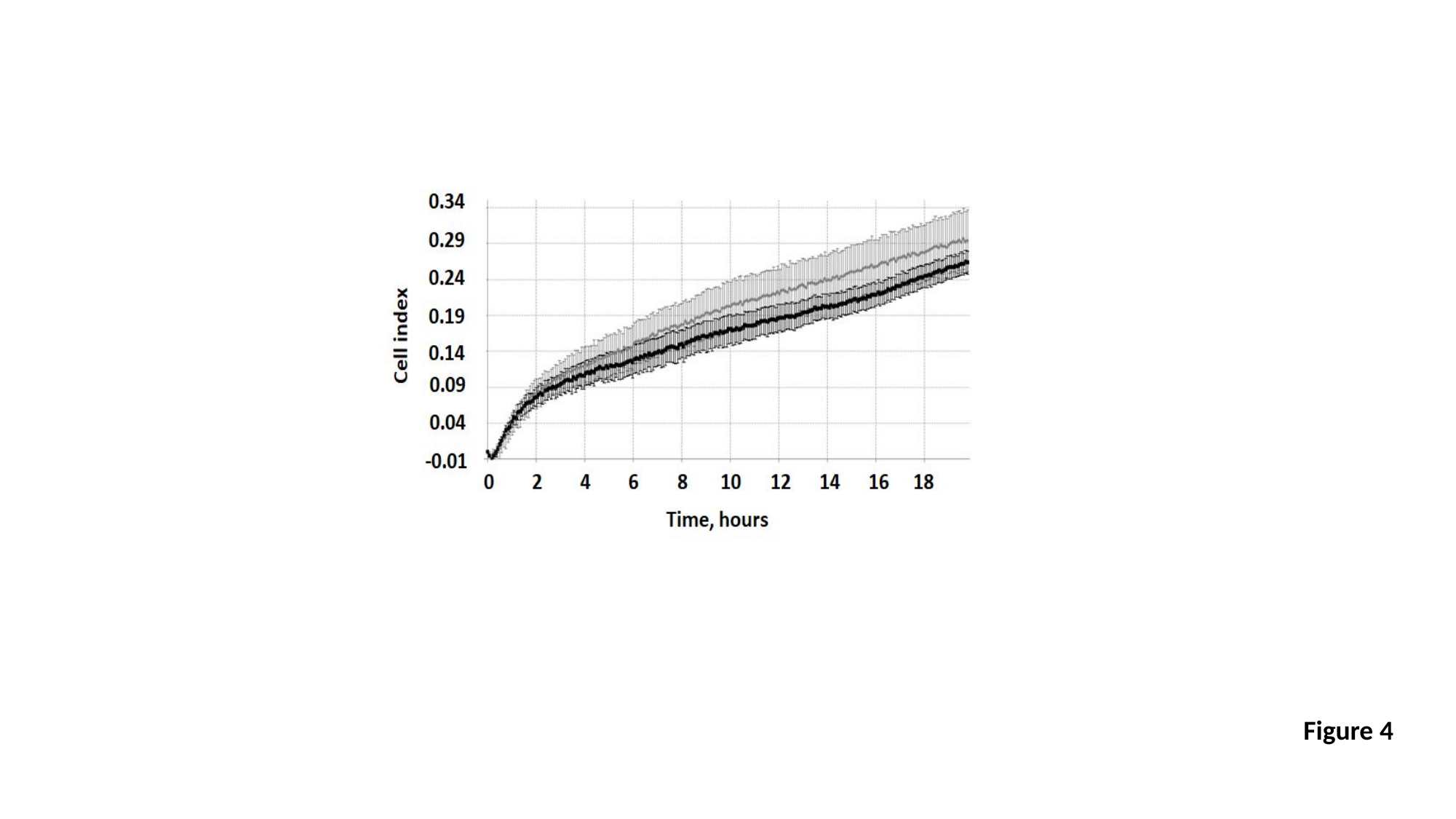

Figure 4

## Slide 5
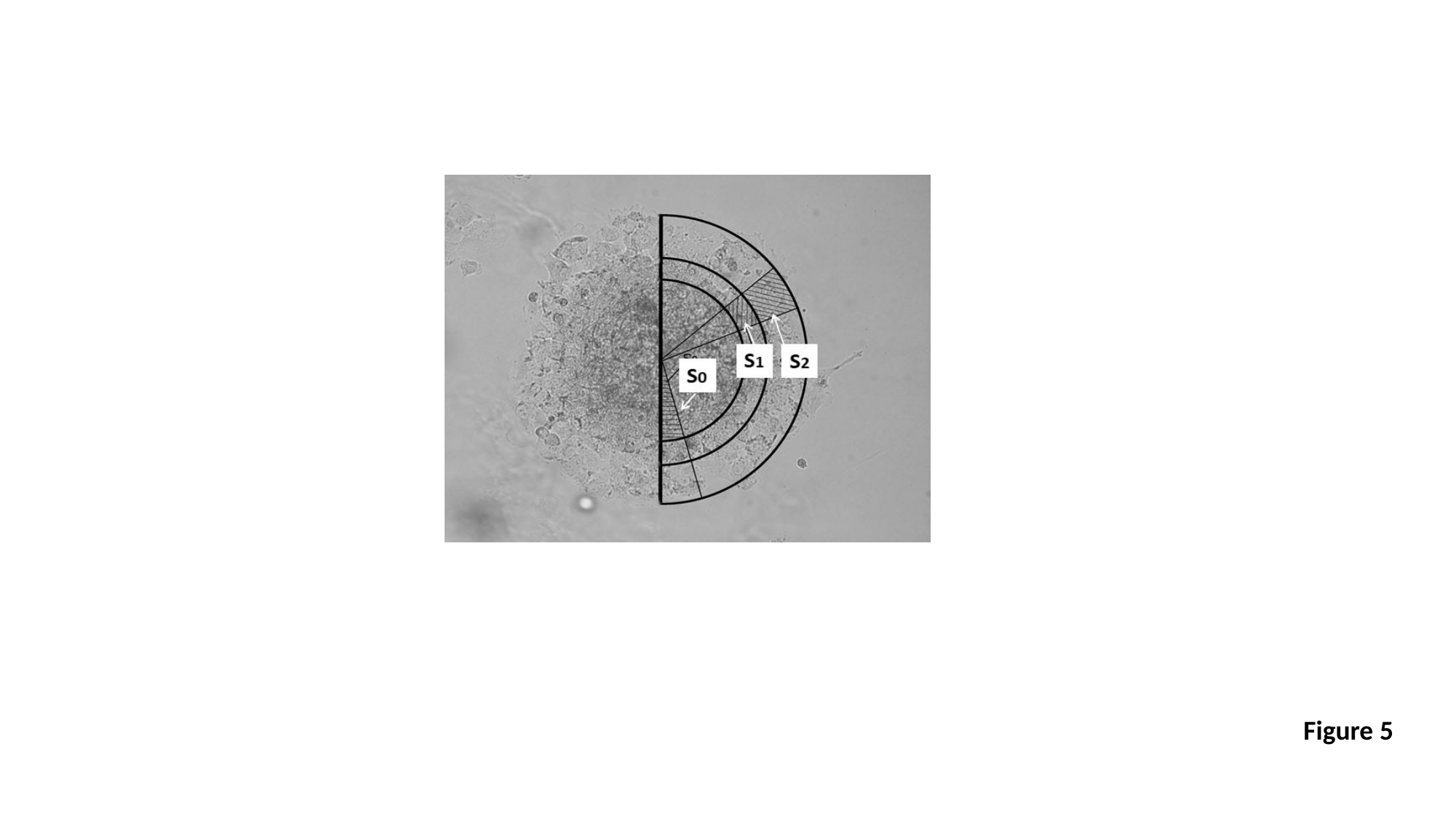

Figure 5

## Slide 6
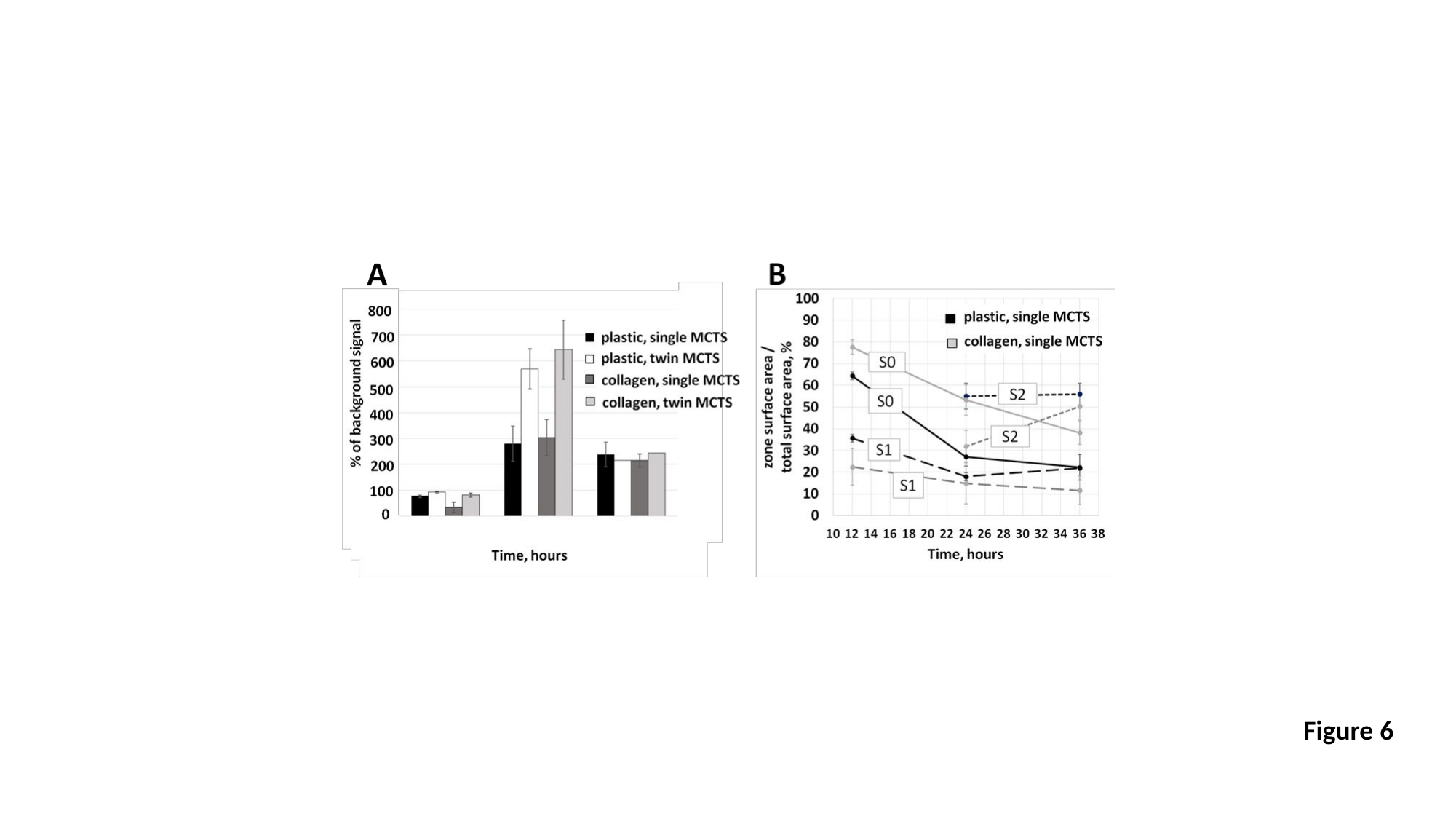

Figure 6

## Slide 7
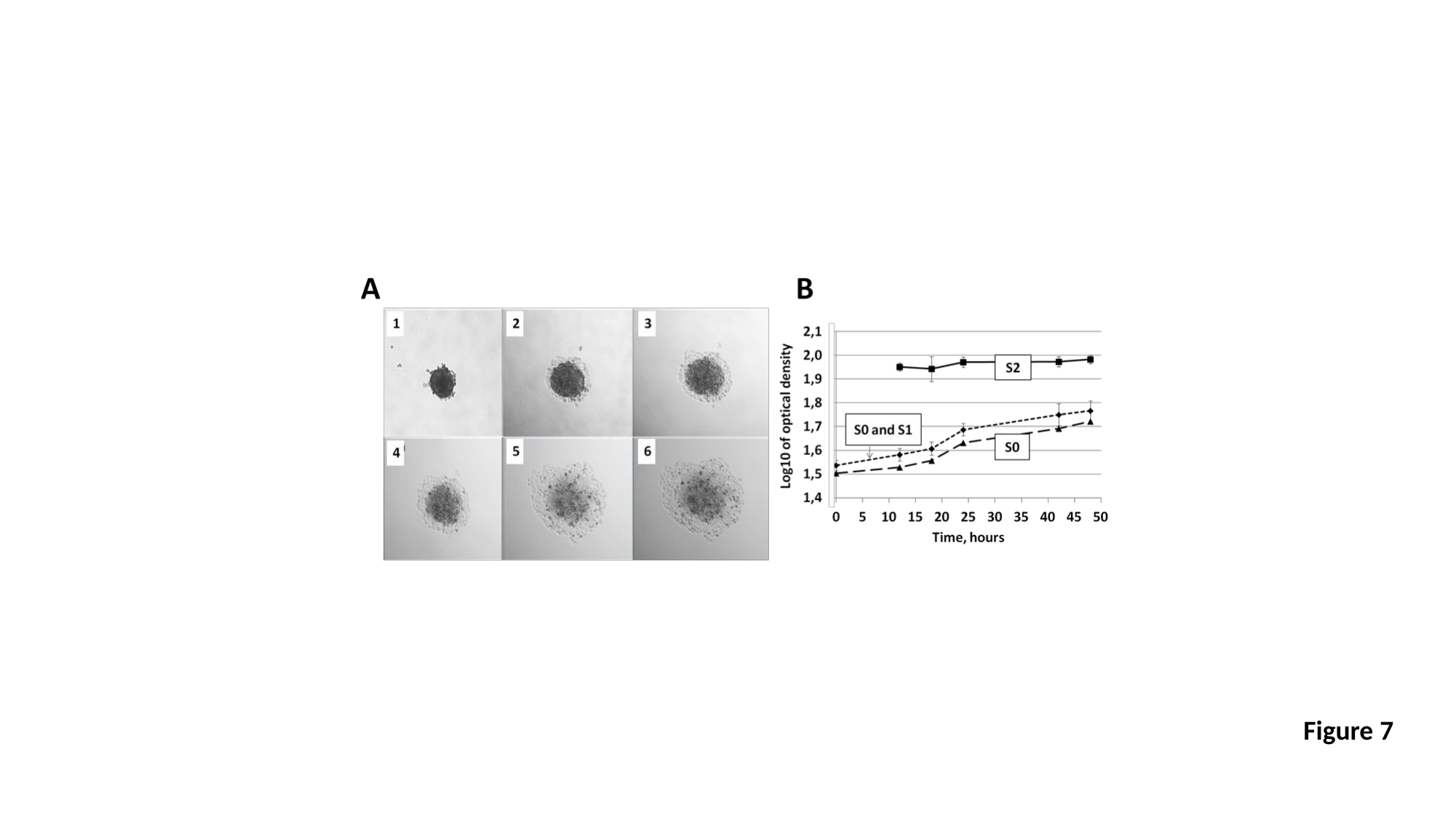

Figure 7

Supplement: Supplementary file 1 [file presentation1.pptx]
